# In Memoriam—Ad. Fellger, M. D.

**Published:** 1888-09

**Authors:** George H. Clark


### IN MEMORIAM.

### AD. FELLGER, M. D.

Adolphus Fellger, M. D., who died on the 19th inst., was born June 14th, 1821, in Gmünd, Wurtemberg, Germany, his parents removing soon after his birth to Stuttgart. In his fifth year he entered the Latin school, having been previously instructed to read and write by a private teacher at home.

Having passed through all his classes, he entered upon the study of medicine under the highly distinguished Dr. Fred. von Hahn. For three years he assisted him in his operations in his surgical clinics, and in his private practice. After passing his examination he attended Paulus College, near Stuttgart, to hear philosophical and philological lectures. He then, although the youngest of thirty-nine candidates, of whom only six were accepted, passed the military surgical examination as first in the class. A few weeks afterward he entered the army as surgeon; becoming during the following winter prosector of anatomy.

He remained in active service for three years, when he took an indefinite leave of absence.

He had then attended medical lectures for six years, witnessing, during that time, the treatment of almost every disease by the most eminent physicians.

By these experiences he suffered such a change in his former high estimate of medical science, that he would have abandoned his profession had he not met with Hahnemann's *Organon*, and witnessed a number of successful cures by means of the homœopathic law, as expounded by that great master of medical science.

He then began to study, with great delight, the works of Hahnemann and other homœopathic physicians, and gradually exchanged the treatment of the old system for that of the new.

After spending two years at the Universities of Tübingen, Zurich, and Strasbourg, he came to this country in 1847.

His life here was a busy one. During the first five years he made a zoological collection, which was presented to the Royal Polytechnic School of Stuttgart. During the late war he was appointed by the German Government to make a report on the military hospitals, for which he received a decoration from the late Emperor William.

Only those who were personally acquainted with Dr. Fellger could fully appreciate his attainments as a man and physician. For he seldom gave to the medical world, through writing, the benefit of his vast experience and his philosophical mind. But what he has written is characterized by profound thought, and shows at once the master's hand.

The practice of Homœopathy was to him a work of love, for he was a homœopathician from principle, and was always able to give the reasons for the "faith that was in him."

In his death our school has met a loss that will not soon be repaired. GEO. H. CLARK.

July 23d, 1888.

